# The Effect of Hydrogen Bonding on Radical Semi-Batch Copolymerization of Butyl Acrylate and 2-Hydroxyethyl Acrylate

**DOI:** 10.3390/polym9080368

**Published:** 2017-08-17

**Authors:** Jan E. S. Schier, David Cohen-Sacal, Owen R. Larsen, Robin A. Hutchinson

**Affiliations:** Department of Chemical Engineering, Queen’s University, 19 Division St, Kingston, ON K7L 3N6, Canada; schier.jan@queensu.ca (J.E.S.S.); 11sdc10@queensu.ca (D.C.-S.); larsen.owen@gmail.com (O.R.L.)

**Keywords:** radical copolymerization, hydrogen bonding, functional comonomer, branching, hydroxyethyl acrylate

## Abstract

The radical copolymerization of butyl acrylate (BA) and 2-hydroxyethyl acrylate (HEA) was investigated under batch and semi-batch operations, with a focus on the influence of hydrogen-bonding on acrylate backbiting. The effect of hydrogen bonding on HEA to BA relative incorporation rates during copolymerization, previously seen in low-conversion kinetic studies, was also observed under high-conversion semi-batch conditions. However, overall reaction rates (as indicated by free monomer concentrations), polymer molar masses, and branching levels did not vary as copolymer HEA content was increased from 0 to 40 wt % in the semi-batch system. In contrast, introduction of a H-bonding solvent, *n*-pentanol, led to an observable decrease in branching levels, and branching levels were also reduced in batch (co)polymerizations with HEA. These differences can be attributed to the low levels of unreacted HEA in the starved-feed semi-batch system.

## 1. Introduction

Radical Polymerization (RP) is one of the most common and significant methods to produce polymers at a commercial scale [1]. Despite the development of controlled radical polymerization [2,3,4], living polymerization [5], and other catalytically supported approaches [6], RP is still favored in industry due to its simplicity and high tolerance towards impurities in either organic or aqueous media [1], and its high productivity at a comparably low cost to produce a diverse range of polymeric products from a wide range of monomers, including ethylene, (meth)acrylates, or styrene derivatives [7].

In the automotive industry, many acrylic based polymers produced by RP are used as coating materials. For optimum processing and reduction of solvent content, these low molar mass polymers must incorporate a certain degree of functionality that is introduced via copolymerization [8]. Functional groups raise the value of the crude product as they allow post-modifications (e.g., crosslinking); whereas, lower molar masses decrease the viscosity when handling or applying the coating to a surface. The reactive functionality is often introduced by hydroxyl containing monomers, from which a wide range of post-modifying chemistry is accessible.

While essential for product application, the addition of functional monomer increases the total polarity and introduces hydrogen bonding to the reaction system, such that solvent choice can exert a significant effect on reaction (propagation) rates [9,10] and copolymer composition [10,11,12]. Recently, we have used a pulsed-laser polymerization (PLP) technique to quantify the influence of solvent on the copolymerization of 2-hydroxyethyl acrylate (HEA) with both butyl methacrylate (BMA) [13] and butyl acrylate (BA) [14]. It was found that HEA incorporates into the copolymer at an increased rate relative to alkyl acrylates, such as BA, in bulk. In addition, unlike alkyl acrylates, the incorporation rate is affected by the choice of solvent, with xylenes promoting HEA incorporation and dimethylformamide (DMF) disrupting its reactivity relative to that in the bulk system.

**Scheme 1:**
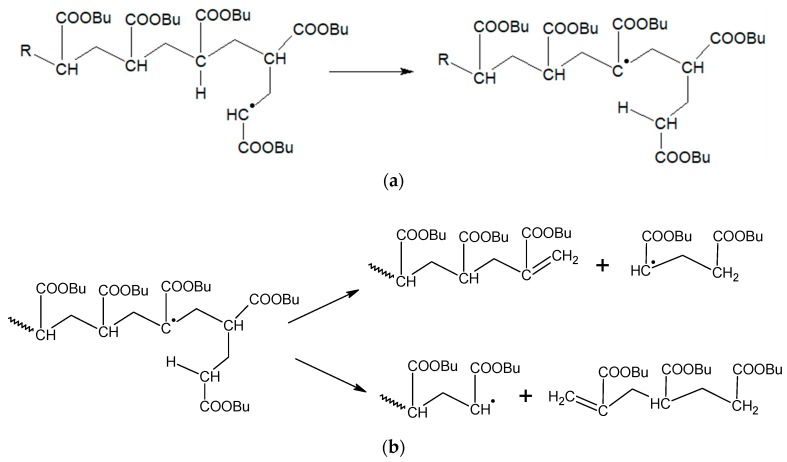
(**a**) Formation of a butyl acrylate (BA) mid-chain radical by intramolecular chain transfer to polymer (backbiting). Monomer addition to the new radical structure creates a short-chain branch in the polymer; (**b**) Macromonomer formation via β-scission of butyl acrylate mid-chain radical.

These specialized PLP studies were conducted at a low conversion, and did not examine the influence of H-bonding on polymer microstructure (branching). It is well-known that propagation of acrylates can be interrupted by the formation of mid-chain radicals by intramolecular chain transfer, in which the secondary propagating radical (SPR) can abstract a hydrogen atom from further along the chain to form a mid-chain radical (MCR) structure, with a favored configuration resulting in a 1,5 H-atom abstraction, as illustrated in Scheme 1a. An important reaction for acrylates [15], a methacrylate unit at the same position does not provide an available H-atom. Therefore, MCR formation is significantly reduced in high methacrylate containing systems [16]. Once formed, the MCR can terminate or undergo two major continuing reactions: propagation, which results in the formation of a branchpoint in the growing polymer chain, and β-scission, which breaks the growing chain into two fragments (Scheme 1b), thus impacting polymer molar mass [8,17,18]. Due to its tertiary nature, the MCRs are more stable than SPRs [19,20], and hence reduce the overall rate of monomer consumption [21]. Indications for β-scission, which becomes a noticeable side reaction at higher temperatures and lowered monomer concentrations, are lower molar mass unsaturated macromonomers, which can be detected by mass spectroscopic techniques [8,18] or by ^1^H NMR [17,22].

Among this sequence of acrylate side-reactions, the rate coefficient for backbiting, *k*_bb_, is of particular importance as it results in the formation of MCRs, which then can further react by termination, propagation, or scission. The rate of polymerization is affected, since the rate of monomer addition to MCRs (*k*_p_^MCR^) is much slower than that to chain-end radicals (*k*_p_), leading to the definition of an averaged coefficient *k*_p_^AV^ [21,23].

(1) $$k_{p}^{\mathrm{AV}}=\;\frac{k_{p}}{1+\frac{k_{\mathrm{bb}}}{k_{p}^{\mathrm{MCR}}\left[M\right]}},$$

Under the assumption that the majority of MCRs form a branching point (valid due to the faster rate of monomer addition to the MCR versus scission and termination rates), a simplified expression relating *k*_bb_ to branching level can be derived, as described by Equation (2). As *k*_p_ has been reliably measured by pulsed-laser polymerization [24], and monomer concentration [*M*] is easily monitored, *k*_bb_ can be estimated provided that the branching level (BL, the fractional quantity of acrylate units that have undergone branching) is accessible through quantitative ^13^C NMR measurements [15].

(2) $$BL\;=\;\frac{k_{\mathrm{bb}}}{k_{p}\left[M\right]+k_{\mathrm{bb}}},$$

This relationship has been used to estimate *k*_bb_ from batch polymerizations of BA in xylenes up to high conversion (90%), between 50 and 70 °C [25], with the resulting values combined with *k*_bb_ estimates from pulsed-laser techniques to yield Arrhenius parameters for the rate coefficient (*A* = 7.4 × 10^7^ s^−1^ and *E_a_* = 32.7 kJ∙mol^−1^); these values were applied by the Hutchinson group to model semi-batch homo-, co-, and terpolymerizations with BA [26,27] up to 170 °C under similar conditions, as examined in this work. However, a recent batch polymerization study, covering temperatures from 60–140 °C proposed that the activation energy is significantly higher [28], indicating the sensitivity of the *k*_bb_ estimates to assumptions made during interpretation of dynamic systems.

Through the presence of branching points from monomer addition to MCRs (which create short-chain branches), or the incorporation of scission-derived macromonomers (which create long-chain branches), a range of random comb-like polymer structures can be formed, possibly affecting the viscoelastic properties of the polymer [29]. While short chain branches do not greatly affect the viscosity, long chain branching points have a significant impact on such physical and application properties [30,31].

The above discussion shows that there is still some uncertainty about branching levels in BA, and data for other acrylate systems is even more scarce. With the introduction of HEA and the associated solvent effects, the influence of H-bonding on acrylate backbiting and related reactions is needed to build on earlier observations of reduced backbiting in such systems [32,33]. Additional conclusions regarding the applicability of low conversion PLP-derived reactivity ratios to industrially relevant operating conditions will be drawn.

## 2. Materials and Methods

Butyl acrylate (BA, ≥99%, Sigma, St. Louis, MO, USA), 2-hydroxyethyl acrylate (HEA, 96%, Sigma), deuterated dimethyl sfoxide (DMSO-d_6_, containing 99.9% D, Cambridge Isotope, Tewksbury, MA, USA), deuterated toluene (containing 99.5% D, Cambridge Isotope), 2,2-azobis(2-methylbutyronitrile) (Vazo-67, >99%, DuPont, Wilmington, DE, USA), *tert*-butyl peracetate (50 wt % in mineral spirits, Sigma), dimethylformamide (DMF, 99%, Sigma), *n*-butanol (BuOH, ≥99.4%, Sigma, St. Louis, MO, USA), *n*-pentanol (PeOH, ≥99%, Sigma), toluene (≥99.5%, Sigma, St. Louis, MO, USA), xylenes (>99%, Fisher Scientific, Hampton, NH, USA), 2-methyl-5-hexanone (methyl isoamyl ketone, MIAK, 99%, Sigma), and butyl propionate (BPi, 99%, Sigma) were used as received without any further purification.

Batch reactions were performed in a 25 mL Schlenk tube under isothermal conditions. Initial monomer to solvent ratios were restricted to 25 vol % monomer to achieve suitable molar masses and branching levels. Vazo-67 was used as an initiator, with an initial concentration of 10 mmol∙L^−1^ based on the total liquid reactor content of 5 mL. In preparation of the reaction, monomer/solvent mixtures were purged thoroughly with nitrogen for at least 15 min, with the subsequent reaction conducted under inert gas conditions at atmospheric pressure for 30 min at 80 °C under constant stirring. The polymerization was then quenched by cooling the tube in liquid nitrogen and the addition of a few drops of a methoxyphenol (2 g∙L^−1^, MEHQ) solution. A portion of the reaction mixture was analyzed via gas chromatography (GC) immediately after the end of reaction to determine monomer conversion. Purification and isolation of the polymer was done by precipitation in a suitable antisolvent, with liquid nitrogen as the cooling agent, such as MeOH/H_2_O (3 to 1 vol ratio) for BA polymers, or diethyl ether for HEA polymers. The crude polymer was then separated from the solution through filtration before drying under vacuum at 60 °C overnight. The exothermic nature of the reaction did not influence reaction conditions, as reaction temperatures did not exceed values higher than 82 °C. Repeat experiments demonstrated that measured conversions were reproducible within a range of 3%, and that molar mass averages within the range of 10%, as shown in the supporting information (Figure S1) for the molar mass distributions measured for poly(BA) synthesized in xylenes.

Semi-batch reactions were conducted in a 0.6 L LabMax reactor system, with automatic stirring, temperature control, a reflux system, and metered reagent mass flow control at 138 °C, a temperature selected as it is the boiling point of xylenes. Other solvents used include ketones (MIAK), esters (BPi), DMF, and pentanol (PeOH). 129 g of solvent was charged to the reactor and heated to reaction temperature under nitrogen inert gas atmosphere. The feed reservoir was charged with 239 g of monomer solution (at various compositions for the case of comonomer systems) before adding 2 mol % of *tert-*butyl peracetate (TBPA) relative to the monomer content, as reported elsewhere [17,27]. The reaction mixture was then fed over 6 h into the reactor while maintaining isothermal operation to reach a final polymer content of 65 wt % in solution, assuming full monomer conversion. Ten samples of 2–3 mL were taken over the course of the reaction. The final end-of-batch samples usually contained more than 10 mL solution to assure proper ^13^C NMR analysis of the polymer isolated. All samples were immediately quenched through the addition of MEHQ inhibitors and stored in the freezer. A fraction was taken to be analyzed via GC for residual monomer concentrations, with residual solvent removed from the remainder of the solvent to isolate the polymer through air stripping and evaporation under vacuum at 60 °C.

Polymers were dissolved in THF at 3–6 g∙L^−1^ and filtered through 0.2 μm nylon filters for size exclusion chromatographic (SEC) analysis of molar mass distributions (MMD). The SEC setup consists of a Waters 2960 separation module connected to a Waters 410 differential refractometer (DRI) (Milford, MA, USA), and a Wyatt Instruments Dawn EOS 690 nm laser photometer multi-angle light-scattering (LS) detector (Santa Barbara, CA, USA). The eluent THF was used at a flow rate of 0.3 mL·min^−1^ through 4 Styragel coloums maintained at 35 °C. The DRI detector was calibrated by polystyrene standards with narrow dispersities over the range of 870–875,000 g∙mol^−1^. The LS detector was calibrated with a single polystyrene (PS) standard as a reference. Based on PS-calibration, absolute molar mass distributions from DRI can be obtained via a suitable transformation using the known Mark-Houwink parameters summarized in Table 1. It is assumed that copolymerization values can be calculated based on the weight-averaged polymer composition, as shown to be valid in previous studies [27,34]. Results were verified by comparing the output from LS detection, which determines absolute molar mass distributions using known refractive indices (*d*_n_*/d*_c_ values).

Monomer concentrations were determined using a Varian CP-3800 GC setup consisting of a CP-8410 autosampler, a CP-117 isothermal split/splitless injector (with a 9:1 split), a 30 M chrompack capillary column (CP-Sil 8 CB), an oven and a flame ionization detector (FID) for quantitative analysis of residual compounds. The separation is based on the different boiling points of the compounds using a fine-tuned temperature profile. Due to the high boiling points above 200 °C of hydroxyl containing monomers, it is recommended to set the injector cell temperature to 275 °C for quantitative evaporation. Accordingly, the separation was optimized with a starting temperature of 100 °C (and a final bake out at 275 °C) for the column oven to avoid line broadening of hydroxyl containing monomers on the stationary phase. Hydroxyl containing monomers elute earlier than their hydrophobic counterparts, despite their higher boiling points. To identify monomers and properly separate them (based on retention time), a calibration was developed with samples of known concentration to construct a plot of an integrated peak area versus monomer concentration. Samples of unknown concentration were prepared by diluting 0.02–0.06 g in roughly 10 g of acetone.

NMR samples were prepared by dissolving dried polymer (free of monomer) in a suitable NMR solvent at concentrations around 2 wt % for ^1^H NMR and significantly higher concentrations of 10–30 wt % for ^13^C NMR to allow for a quantitative detection of the naturally less abundant ^13^C-nuclei. Nonpolar homopolymers were commonly analyzed in CDCl_3_, whereas hydroxyl functional copolymers were analyzed in DMSO-d_6_. Two different Bruker Avance spectrometers were used at 400 or 500 MHz. ^1^H NMR spectra were recorded at standard conditions with 3.17 s acquisition time, 1 s relaxation delay, 6 μs dead time, and 48.4 μs dwell time. The quantitative ^13^C NMR was acquired under inverse gated WALTZ16 conditions to suppress the Nuclear Overhauser related effects (NOE) with a sufficient main delay time of 10 s per scan, as reported elsewhere [15,17]. A sufficient signal to noise ratio was achieved after 3000 scans, with run times generally adjusted to allow for more than 4000 scans. Supporting analysis via 2D and DEPT NMR methods can be found elsewhere [35].

## 3. Results and Discussion

### 3.1. Batch Experiments

A series of batch experiments were performed to examine the influence of H-bonding on backbiting in acrylate polymerization under time-varying monomer concentration profiles, providing a point of comparison to the semi-batch polymerizations conducted at low and relatively constant monomer concentrations. The initial monomer content (25 vol %) was kept low to eliminate any potential influence of increasing viscosity on polymerization rate, and reaction temperature (80 °C), initiator concentration (10 mmol·L^−1^), and reaction time (30 min) were also kept constant for the various acrylate/solvent combinations studied under batch conditions. As summarized by Table 2, the monomer conversions reached in 30 min ranged from 76% to 90%. As final conversions are similar, the resulting BL values measured provide insights as to how solvent and monomer choice affects the relative rate of backbiting to chain-growth (*k*_bb_/*k*_p_).

Monomer conversions of 75–80% were reached for BA polymerized in xylenes and toluene, with the polymer produced in both solvents having a very similar number average (*M*_n_) and weight-average (*M*_w_) molar masses, as well as final branching levels (~3%). The influence of H-bonding can be seen when comparing these results to the polymerization of BA in BuOH; significantly lower BLs are measured (2.4%), despite the slightly higher conversion (85%) attained. Furthermore, the polymer molar masses are higher for the polymerization in BuOH when compared to toluene and xylenes. These results are consistent with the differences previously reported for semi-batch polymerization of BA in BuOH when compared to xylenes [32], as well as kinetic studies that have shown that the use of alcohol as a solvent significantly increases *k*_p_ for alkyl (meth)acrylates [13,36], while also decreasing acrylate *k*_bb_ [32,33]. It is hypothesized that H-bonding between the alcohol solvent and polymerized-BA units sterically hinders chain-end movement, such that the occurrence of the 1,5 backbiting mechanism is reduced relative to chain growth. Supporting this suggestion, infrared spectroscopy has confirmed H-bonding between carbonyl groups on a poly(BA) chain and BuOH [37], similar to that observed between unreacted (meth)acrylate monomers and hydroxyl groups [13,38]. The resulting higher fraction of chain-end radicals in the system leads to higher rates of polymerization and polymer molar masses in the system; as a secondary effect, the longer polymer chains will lead to a decrease in the termination rate coefficient [39,40], further increasing rate and polymer molar masses.

To enhance this knowledge about solvent-induced H-bonding effects, experiments were also carried out with HEA, in both butanol and DMF (which disrupts H-bonding). As poly(HEA) is sparingly THF-soluble, only conversion and branching data are available. Monomer conversions were found to be similar or slightly higher than those of BA, 90% in BuOH and ≈85% in DMF. The branching level was 1.2% for poly(HEA) synthesized in BuOH, half that measured for poly(BA) in BuOH, and decreased even further relative to BA polymerized in xylenes and toluene. Thus, the combined influence of H-bonding induced by solvent and monomer further reduces the occurrence of backbiting in the system.

A previous PLP low-conversion study found that branching levels in poly(HEA) increased for polymerization in DMF relative to bulk, but the extent of the increase was not quantified [33]. The 1.8% level of branching measured in this work for batch polymerization of HEA in DMF is higher than that found in BuOH, but is still significantly lower than poly(BA) branching levels. A possible explanation is that the interaction of DMF with polymerized HEA units also stiffens polymer chains to reduce branching. Further insights may be gained by examining the correlation of branching with polymer-solvent and polymer-monomer interactions, as quantified by Hansen solubility parameters [41], or Kamlett-Taft solvatochromic parameters [42].

The batch experiments indicate that backbiting rates are reduced for BA in the presence of a H-bonding solvent, and are always decreased for HEA as a monomer. It is of interest, then, to see the influence of HEA added as a comonomer to the extent of acrylate branching. BA/HEA batch copolymerizations were conducted in both xylenes and MIBK. Despite the high solvent content, both reactions underwent phase separation (precipitation of polymer) after roughly 15 min, with the heterogeneity occurring in xylenes earlier than in MIBK. It is known that HEA preferentially incorporates into the copolymer [14], which likely contributes to the insolubility as the monomer concentration is depleted. Thus, it is more difficult to evaluate the branching results, which are of the same level as observed for HEA homopolymerization. However, as the monomer conversions are considerably lower at the time of precipitation, it suggests that the branching rates (relative to propagation) are higher for copolymerization than for HEA homopolymerization. To obtain better control in these industrially relevant solvents, the next section presents results for semi-batch copolymerization, with increased HEA contents and smaller target polymer molar masses.

### 3.2. Semi-Batch Copolymerization of BA and HEA in Various Solvents

**Figure 1:**
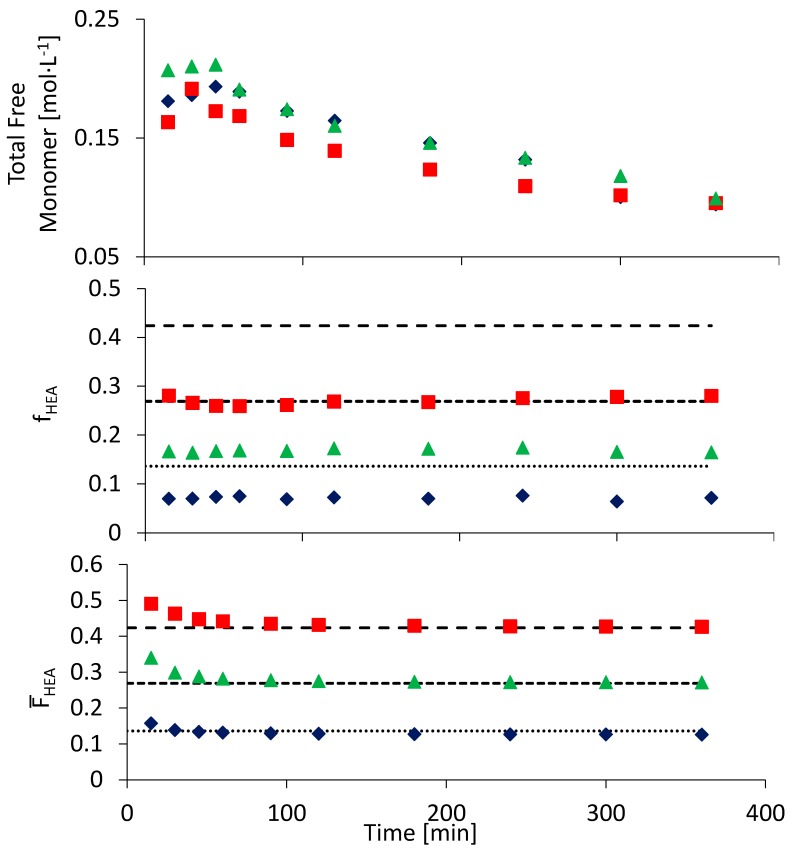
Total free monomer concentration (**top**), HEA molar fraction in the comonomer (*f*_HEA_) (**middle**) and copolymer (*F*_HEA_) (**bottom**) for BA/HEA semibatch copolymerizations in esters (BPi) at HEA levels of 12.5 wt % (♦), 25 wt % (▲), 40 wt % (■) in the comonomer feed.

The semi-batch experiments were performed under conditions commonly used in industry [8,27], with a six-hour monomer feeding period, 2.0 mol % initiator relative to monomer, a reaction temperature of 138 °C, and a final polymer content of 65 wt % in solution. Due to its more polar nature (in comparison to xylenes), BPi was chosen as a solvent to ensure good solubility over a wide range of HEA compositions [35]. Experiments could be performed up to HEA levels of 40 wt %, with no phase separation or precipitation observed under reaction conditions; experiments with higher HEA content were not conducted as some evidence of precipitation was observed with 50 wt % HEA. The change in total free monomer content as a function of reaction time is shown in Figure 1 (top). Independent of the HEA content, the concentration of total free monomer ([BA] + [HEA]) remains roughly constant, decreasing from an initial level of ~0.2 to less than 0.1 mol∙L^−1^ over the course of the 6 h period of monomer feeding, with perhaps slightly lower monomer levels seen for the run performed with 40 wt % HEA. These low monomer levels are characteristic of starved-feed semi batch operations, as the instantaneous monomer conversions are high due to the fast polymerization rates, and the decrease in concentration over time a result of the monomer being fed at a fixed flowrate into an increasing reaction volume [8,27].

Figure 1 also plots the molar fraction of HEA in the monomer, *f*_HEA_, as calculated from the GC data, with the corresponding values of copolymer composition, *F*_HEA_, calculated by mass balances. After an initial transient period, constant polymer and monomer compositions are maintained over the course of the feeding period. As expected for a starved-feed operation, the HEA fraction in the copolymer quickly attains the target (feed) value, denoted by the horizontal dotted lines on the plot. However, the HEA monomer fraction plateaus at values significantly below the molar feed composition. The observation that *f*_HEA_ < *F*_HEA_ in the system is consistent with the low conversion PLP experiments [14], as there is an increased relative reactivity of HEA relative to BA.

**Figure 2:**
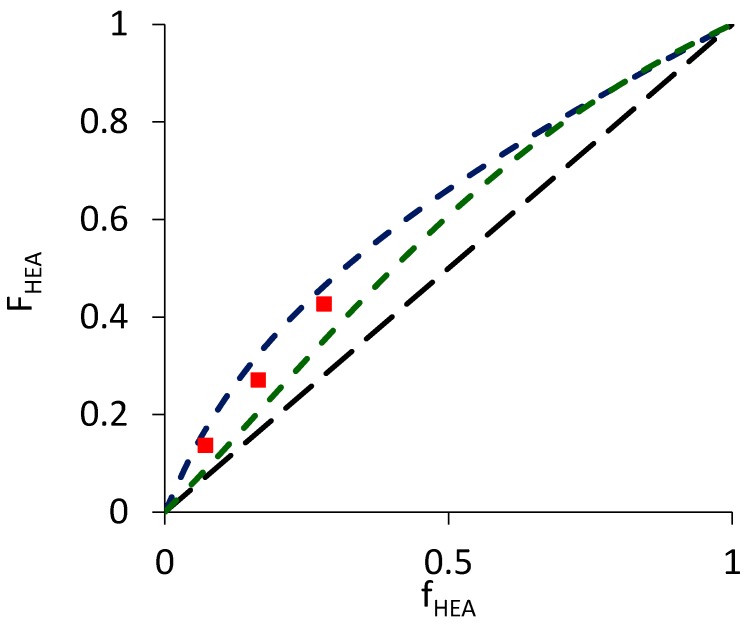
Comparison of HEA copolymer mole fraction (*F*_HEA_) as a function of HEA comonomer mole fraction (*f*_HEA_) under semi-batch operating conditions for BA/HEA copolymerizations in BPi (■) to terminal model fits to data obtained by low conversion kinetic studies in bulk (**---**) and methyl isobutyl ketone (**---**) [14].

Under these semi-batch operating conditions, the concentration of HEA in the reactor is always less than 0.05 mol∙L^−1^ during the entire course of the reaction, and generally much lower. Even for the experiment with 40 wt % HEA in the feed, a total free monomer of 0.15 mol∙L^−1^ and *f*_HEA_ of 0.28, results in [HEA] = 0.04 mol∙L^−1^. These conditions are very different than the high monomer concentration conditions used in the PLP study of HEA-BA copolymerization chain-growth kinetics [14], in which HEA was found to be preferentially incorporated into the copolymer. The relationship between comonomer and copolymer composition data of each semi-batch run (as determined from the final several hours of operation) is compared with the PLP derived Mayo-Lewis plot for BA/HEA in Figure 2. The semi-batch data (albeit limited) fall between the terminal model and fits to low-conversion copolymer composition data for BA/HEA in bulk and in ketones, which have a similar effect on composition as BPi [14,35]. Therefore, the chemical environment during the semi-batch process still leads to the preferential incorporation of HEA into the copolymer at a level that is reasonably described by reactivity ratios obtained from kinetic studies at higher monomer concentrations and lower temperatures.

**Figure 3:**
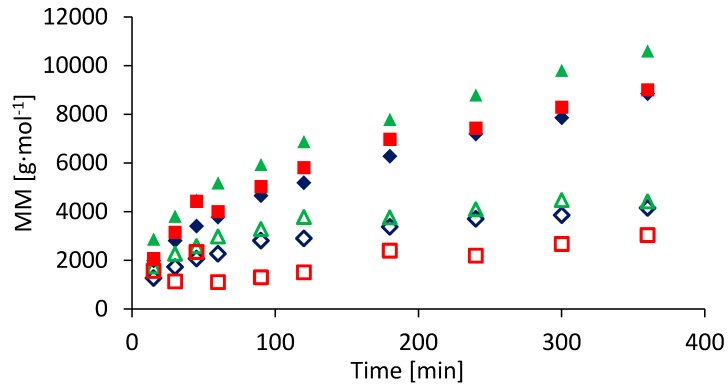
Evolution of number- (open symbols) and weight- (filled symbols) average polymer molar masses for BA/HEA copolymer produced via semi-batch polymerization in BPi with 12.5 (♦), 25 (▲) and 40 wt % (■) HEA in the feed.

The analysis of copolymer composition indicates that the presence of HEA in the recipe promotes its relative incorporation rate, despite the low concentration of free monomer under semi-batch operating conditions. Still to be examined is its influence on polymer properties. As shown in Figure 3, the effect of substituting HEA for BA in the acrylate recipe has only a minor effect on polymer molar masses; the evolution of number and weight MM averages over time are similar with 12.5, 25, and 40 wt % HEA in the acrylate mixture, with the final values very similar to those measured for BA homopolymerizations in BPi (*M*_n_ = 4000 and *M*_w_ = 8600 g∙mol^−1^). The general shape of these curves, with poly(acrylate) MW values increasing over time, has been explained by the incorporation of macromonomers into the growing polymer chains [22]. Thus it can be generalized from this set of HEA/BA copolymerizations in BPi that, although the HEA is preferentially incorporated into the polymer, its addition to the recipe has little effect on overall reaction rates (total free monomer levels), and the molar masses of the polymer product. When adding HEA to the recipe, however, the solvent must have sufficient polarity to prevent polymer precipitation. In BPi, 40 wt % HEA in the copolymer could be easily synthesized at 138 °C, but 50 wt % HEA was too high [35].

Semi-batch BA/HEA copolymerizations with 25 wt % HEA in MIAK and xylenes were also conducted, using the same reactor conditions and following the same monomer and initiator feeding schedules as used with BPi. Free monomer concentrations and comonomer compositions were of a similar magnitude as for the reaction in BPi, showing a significant promotion of HEA incorporation through H-bonding. As found in BPi, the addition of HEA to the recipe did not greatly impact the polymer molar masses when compared to BA homopolymerization (Figure S2 in Supporting Information). Small differences found in the MMDs are attributed to the differences in transfer to solvent reactions, as the polymer *M*_w_ values produced for all recipes in BPi were consistently higher by 1000–2000 g∙mol^−1^ than for polymers produced in xylenes [35].

Further copolymerizations of BA and HEA (with 25 and 50 wt % HEA) were carried out in DMF and PeOH solution to develop more insights about the effects of H-bonding under semi-batch conditions; PeOH was used instead of BuOH to keep below the solvent boiling point at 138 °C. Monomer concentration and composition data are summarized in Figure S3, with corresponding polymer MM averages plotted as Figure S4. For these cases, the total monomer concentrations were also less than 0.1 mol∙L^−1^ throughout much of the reaction, although the levels were unexpectedly higher (>0.3 mol∙L^−1^) for the first hour of HEA/BA copolymerization in PeOH. This difference in initial transient behavior was accompanied by a high fraction of HEA in the monomer mixture, and may be an indication that the relative incorporation of HEA and BA in alcohol solvent is concentration dependent, as observed for low conversion studies of HEMA copolymerized with styrene [38]. Nonetheless, the fraction of HEA in the comonomer quickly settled to a constant level value after 1 h, with *f*_HEA_ values summarized along with all other experiments in Table 3. In the low conversion studies of this system, it was found that the copolymer composition data was close to the diagonal for reactions in BuOH and in DMF [14], indicating very similar BA and HEA relative reactivities in these two solvents. Under the semi-batch conditions of this study, however, HEA is preferentially incorporated (*F*_HEA_ > *f*_HEA_). Thus, the presence of the functionalized copolymer (65 wt % in final solution) promotes the HEA incorporation to a level intermediate between that predicted using reactivity ratios derived from low-conversion bulk and solution experiments. The polymer *M*_w_ values produced in PeOH and DMF solutions are significantly lowered when compared to the values measured in the other solvents, in agreement with other literature that indicates these solvents are more prone to H-atom abstraction [43,44,45]. A similar decrease was seen in the analysis of BA semi-batch homopolymerization in DMF when compared to xylenes in a previous study [12].

### 3.3. Acrylate Backbiting under Semi-Batch Conditions

Transfer to polymer reactions result in either short-chain (SCB) or long-chain (LCB) branchpoints, with the subsequent slow addition to midchain radicals (MCR), decreasing polymer chain growth rates, and the scission of MCRs decreasing polymer molar masses. Although LCBs influence rheological performance, their level in poly(acrylate) systems is low, with the majority of branching occurring by backbiting to form SCBs [22,23,25]. The branching levels (BLs) reported in Table 3 have been determined for the final samples from the set of semi-batch reactions via quantitative ^13^C NMR by measuring the amount of quaternary carbons (peak position at 48 ppm) relative to the total acrylate units in the polymer chain, as shown by the example spectrum in Figure S5 and as based on previous literature studies [15,17,28].

**Figure 4:**
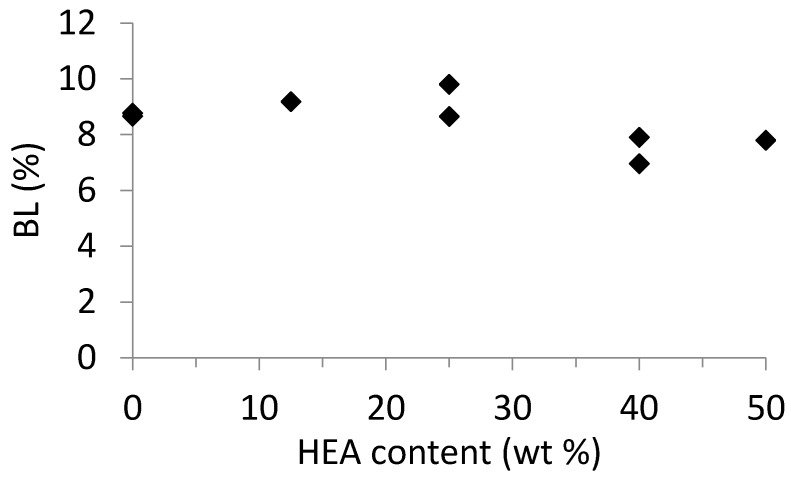
Polymer branching levels (BL) as a function of HEA content (wt %) in the comonomer feed, measured for poly(acrylate) copolymer produced by semi-batch BA/HEA copolymerizations in BPi solvent at 138 °C.

One important question examined in this study is how an increase in HEA fraction in the feed influences the level of branching (and hence *k*_bb_), as previous work has shown that H-bonding induced by alcohols lowered BLs during semi-batch homopolymerization of BA at 110 °C [32]. Figure 4 presents the BLs measured for semi-batch BA/HEA copolymerizations in BPi at 138 °C, with HEA levels up to 50 wt %. Between 7–11% of the acrylate units in the polymer are quaternary, much higher than measured for the solution batch reactions (see Table 2), a difference due to the higher temperature and the lower free monomer levels in semi-batch operation. These levels are similar to those reported for investigations of semi-batch BA systems in xylene [12,17], indicating that BPi as a solvent does not greatly influence the rate of backbiting. Remarkably, there is no significant decrease in the measured BL with increasing HEA levels incorporated into the copolymer.

The finding that HEA reduces acrylate BLs in batch (Table 2) but not semi-batch (Figure 4) can be explained by considering the differences in reaction environment between the two systems. Under batch conditions, the level of unreacted HEA is much higher throughout much of the reaction when compared to the semi-batch system, in which HEA concentrations are always below 0.1 mol∙L^−1^. Thus, these results suggest that a threshold concentration of the H-bonding species is required to significantly reduce the rate of backbiting. This interpretation also explains the major difference between this HEA/BA semi-batch system, and the previous reduced BLs found with poly(BA) produced by semi-batch in an alcohol solvent [32]. In the latter system, the H-bonding is provided by the solvent, which remains abundant over the entire course of the reaction. Already supported by infrared spectroscopic measurements [37], a comparison of the size of polymer chains (e.g., by dynamic light scattering [46]) in the presence and absence of alcohols could provide additional insights to the stiffness (conformation) of polymer chains induced by H-bonding.

The results shown in Figure 4 imply that the HEA/BA copolymer backbone remains as flexible as a BA homopolymer within a BPi solution with low HEA content, allowing for the necessary H-abstraction from the 1:5 position, and in contrast to the presumed rigidity induced with higher concentrations of H-bonding species (either solvent or monomer) in solution. This difference is seen by comparing to BLs measured for the semi-batch copolymerizations in PeOH, also summarized in Table 3. The branching levels measured in PeOH were in the range of 6–7%, consistently lower than the 8–9% levels measured in DMF and in BPi. For all solvents, the level of branching did not vary significantly with copolymer composition.

A simple comparison of BLs between the systems, however, can be misleading, as the quantity is a function of monomer concentration and propagation kinetics, as well as the rate of backbiting. Thus, the data were used in the following rearrangement of Equation (2) to calculate the ratio of *k*_bb_ (more precisely, *k*_bb_^cop^) to *k*_p_^cop^ according to:

(3) $$\frac{k_{\mathrm{bb}}}{k_{p}^{\mathrm{cop}}}=\;\frac{BL\;\cdot \;\left[M\right]}{\left(1-BL\right)}$$

As total monomer concentration and composition do not vary greatly over the course of the semi-batch reactions, the value of [*M*] is taken as an integrated average over the whole reaction time, as summarized in Table 3. Even though Equation (3) assumes that all backbiting events lead to formation of a branchpoint, neglecting the consumption of MCRs by competing scission and termination reactions, as well as the fact that growing chains of length less than four units cannot undergo backbiting, the normalization provides a reasonable means of comparison between the systems. For example, while the BL measured in xylenes is lower than those measured in MIAK and BPi (0.07 as compared with 0.09–0.10) for experiments conducted with 25 wt % HEA, the final estimates for *k*_bb_/*k*_p_^cop^ were in the same range of 0.014–0.016 L∙mol^−1^.

The estimate for *k*_bb_/*k*_p_^cop^ decreases from 0.019 L∙mol^−1^ for BA homopolymerization to 0.013–0.014 L∙mol^−1^ for HEA/BA copolymerizations at identical semi-batch reaction conditions in BPi. The decrease indicates an increase in the value of *k*_p_^cop^ with the addition of HEA to the recipe, a decrease in *k*_bb_, or a combination of the two. As the PLP-SEC study determined that *k*_p_^cop^ increased by 20–30% as *f*_HEA_ increased from 0 to 0.3 under low conversion conditions [14], it is likely that the major factor is an increase in chain-end reactivity, caused by the addition of HEA, although a small decrease in *k*_bb_ cannot be ruled out. The influence of PeOH, present at much higher concentrations than HEA under starved-feed semi-batch conditions, is more significant, reducing *k*_bb_/*k*_p_^cop^ by close to a factor of 2 when compared to values found in the other solvents at 138 °C. This decrease is lower than the factor of 4–5 reduction reported in a previous BA homopropagation investigation at 110 °C by Liang et al. [32]. Liang also compared branching levels for BA in xylene and pentanol at 138 °C in his PhD thesis [37], for which he found that branching levels decreased roughly by the same factor of two as found here. Thus, the influence of H-bonding diminishes with increased temperature, a result in agreement with the hypothesis that it is the chain stiffness induced by the interaction of the H-bonding solvent with reacting chain ends that reduces backbiting.

Finally, the results obtained in DMF warrant discussion. Although the absolute branching levels are in the same 9% range as observed in other solvents, the reduced free monomer levels lead to lower estimates of 0.010–0.012 L∙mol^−1^ for *k*_bb_/*k*_p_^cop^. Whereas in PeOH the effects are clearly derived from H-bonding, the reasoning for DMF is complicated, due to multiple possibilities of the polar or mesomerically charged DMF to affect the reactivity during the semi-batch copolymerization process. However, the trends agree with the findings from small-scale batch experiments, which showed reduced branching levels for both BA and HEA in DMF (Table 2).

**Figure 5:**
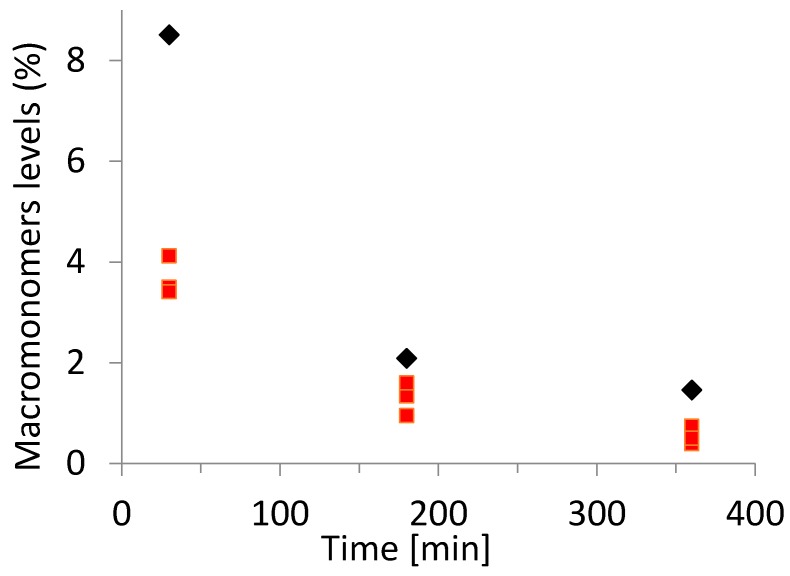
Macromonomer content (per 100 acrylate repeat units) determined by ^1^H NMR, measured in polymer samples taken during semi-batch polymerization of BA (♦) and BA/HEA (■) in BPi at 138 °C.

As shown in previous investigations, the BL is closely associated with the rate of chain scission in the system due to the competition between monomer addition (leading to the formation of a quaternary carbon) and scission (resulting in the formation of a chain with an unsaturated double bond, or macromonomer) of the MCR formed by backbiting. Figure 5 shows the evolution of macromonomer levels over the course of the semi-batch reactions in BPi, determined by measuring the presence of vinyl peaks in ^1^H NMR spectra, as shown in Figure S6. The general shape of the curve, with decreasing macromonomer levels with increasing time, is controlled not only by the generation rate of macromonomers through scission, but also by their consumption rate by reaction, as detailed in the study by Wang et al. [22]; the macromonomer levels shown in Figure 5 for poly(BA) are in good agreement with that previous work. The presence of HEA in the polymer reduces the level of unsaturated double bonds independent of the HEA content, even though the *M*_n_ values of the HEA-containing polymers are lower than that of poly(BA) (see Figure 3). Whereas an exact mechanistic explanation cannot be given, it may be that the presence of HEA in the backbone allows for interactions with free monomer or solvent that provides some stabilization and inhibits scission of an MCR.

## 4. Conclusions and Outlook

The investigation of acrylate-only copolymerization of BA and HEA through batch and semi-batch experiments has provided additional insight into the effect of H-bonding on copolymerization kinetics. A distinction can be made between the effects induced by hydroxyl functional monomers themselves, and alcohol as a solvent. It was found that the hypothesis for an increased relative incorporation rate of HEA through H-bonding in copolymerization, derived by PLP experiments, withstands operational changes towards batch and semi-batch reactions up to temperatures of 138 °C. Though still present in the high solids content semi-batch system, the solvent effects on composition were not as pronounced as in PLP low conversion experiments, with copolymer compositions found to be closer to those expected for a bulk system.

The influence of H-bonding on the acrylate side reactions of backbiting and scission was also investigated. It was verified that H-bonding reduces the level of branching in acrylate semi-batch polymerization in alcohols. While H-bonding introduced by HEA causes a similar reduction in branching levels during batch polymerization, the concentration of HEA is too low to reduce the backbiting rate under semi-batch conditions, as the HEA content within the copolymer had no notable effect on the *k*_bb_/*k*_p_^cop^ values estimated in BPi, xylenes, and MIAK. In contrast, scission was found to be comonomer dependent, with reduced macromonomer levels present in HEA copolymers. While further work is required to fully explain these phenomena, the experimental study shows clearly that adding HEA to an acrylate-only copolymerization system does not greatly impact the rates and polymer properties (molar masses and branching levels) under typical semi-batch operating conditions, provided that a suitable solvent for the polar copolymer system is chosen.

## Figures and Tables

**Table 1:** Parameters required for analysis of polymer molar mass averages and distributions.

Monomer	*d*_n_/*d*_c_ (mL⋅g^−1^)	Mark-Houwink Parameters in THF	
		*K* (dL⋅g^−1^)	*a*
HEA [34]	0.066	0.000322	0.602
BA [24]	0.064	0.000122	0.700
Styrene [34]	0.184	0.000114	0.716

**Table 2:** Final monomer conversions, average polymer molar masses and branching levels measured for poly(acrylates) produced via 30 min batch polymerizations conducted at 80 °C with 75 vol % solvent.

Monomer	Solvent	[*M*]_ini_ (mol∙L^−1^)	Conversion (%)	*M*_n_/*M*_w_ (g∙mol^−1^)	Branching Level ^b^ (% per Acrylate Unit)
BA	Xylenes	1.65	80.7	21,100/41,100	3.1
BA	Toluene	1.65	75.9	17,100/39,500	3.0
BA	*n*-Butanol	1.65	84.2	36,800/87,700	2.4
HEA	DMF	2.25	84.8	n.a.	1.8
HEA	*n*-Butanol	2.25	90.0	n.a	1.2
BA/HEA^a^	Xylenes	1.90	Phase separation/precipitation occurred before 30 min; *F*_HEA_ >> *f*_HEA_ at conv. ≈ 50%		1.2
BA/HEA^a^	MIBK	1.90			1.8

^a^ BA/2-hydroxyethyl acrylate (HEA) copolymerizations were performed at a 50/50 *vol*/*vol* monomer ratio; ^b 13^C NMR techniques are reliable above branching levels of 1% per acrylate unit and are associated with an absolute error of ±0.5%.

**Table 3:** Copolymer compositions, polymer properties, and estimated backbiting rate coefficients ratioed to propagation (*k*_bb_/*k*_p_^cop^) from BA/HEA semi-batch copolymerizations conducted in various solvents at 138 °C.

Comonomer Feed (wt %) and Solvent	*F*_HEA_^a^	*f*_HEA_^a^	*M*_w_^b^ (g∙mol^−1^)	BL ^b^	∫[*M*] ^c^ (mol∙L^−1^)	*k*_bb_/*k*_p_^cop^ (L∙mol^−1^)
BA (100) BPi	0	0	8600	0.088	0.203	1.95 × 10^−2^
BA/HEA (87.5/12.5) BPi	0.13	0.07	8900	0.092	0.141	1.43 × 10^−2^
BA/HEA (75/25) BPi	0.27	0.17	9000	0.087	0.146	1.38 × 10^−2^
BA/HEA (60/40) BPi	0.43	0.28	10,600	0.092	0.125	1.27 × 10^−2^
BA/HEA (75/25) MIAK	0.27	0.16	7100	0.098	0.151	1.64 × 10^−2^
BA/HEA (75/25) xylenes	0.27	0.15	7600	0.072	0.193	1.49 × 10^−2^
BA PeOH	0	0	3900	0.064	0.113	0.78 × 10^−2^
BA/HEA (75/25) PeOH	0.27	0.14	4400	0.067	0.152	1.09 × 10^−2^
BA/HEA (50/50) PeOH	0.53	0.32	3400	0.071	0.110	0.84 × 10^−2^
BA/HEA (75/25) DMF	0.27	0.20	4000	0.095	0.111	1.17 × 10^−2^
BA/HEA (50/50) DMF	0.53	0.41	3800	0.093	0.099	1.01 × 10^−2^

^a^ Steady-state values of mole fraction HEA in copolymer (*F*_HEA_) and comonomer (*f*_HEA_) attained after initial 1 h transient period; ^b^ values measured for final polymer samples; ^c^ indicates a time-averaged value of total monomer concentration over the 6 h reaction time.

## Supplementary Materials

Additional figures (as discussed in the text) are online at www.mdpi.com/2073-4360/9/8/368/s1.
