# Supplementary material for: The Effect of Hydrogen Bonding on Radical Semi-Batch Copolymerization of Butyl Acrylate and 2-Hydroxyethyl Acrylate
Source: Polymers (Basel). 2017 Aug 17;9(8):368. doi: 10.3390/polym9080368 (PMC6418611; doi:10.3390/polym9080368)
Supplement: Supplementary file 1 [file polymers-09-00368-s001.pdf]

Supporting Information

The effect of hydrogen bonding on radical semi-batch copolymerization of butyl acrylate and 2-hydroxyethyl acrylate

Jan E. S. Schier, David Cohen-Sacal, Owen R. Larsen, Robin A. Hutchinson*

Department of Chemical Engineering, Queen's University, 19 Division St, Kingston, ON, K7L 3N6 CANADA. E-mail: robin.hutchinson@queensu.ca

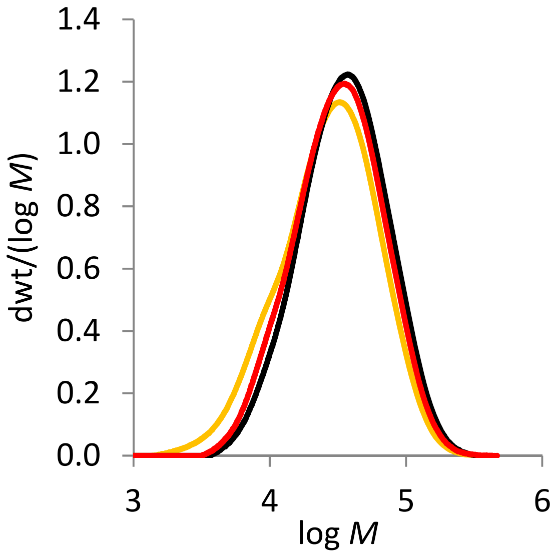

Figure S1: MMDs of three poly(BA) samples produced by 30 min batch reactions of 25 v% BA in 75 v% xylenes, illustrating excellent reproducibility of the experimental procedures.

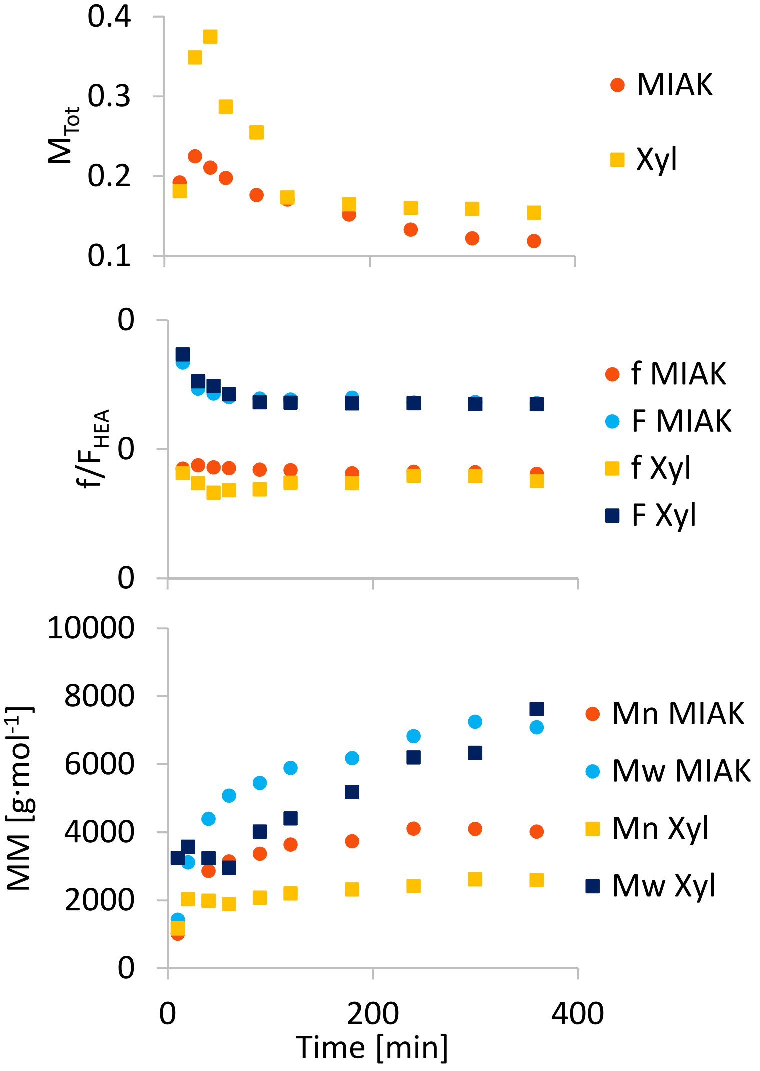

Figure S2: Evolution of total free monomer (top) and number-average (M_n) and weight-average (M_w) polymer molar masses (bottom) formed by copolymerization of BA/HEA with 25 wt% HEA in the feed under semi-batch conditions at 138 °C in xylenes (Xyl) and methyl isoamyl ketone (MIAK).

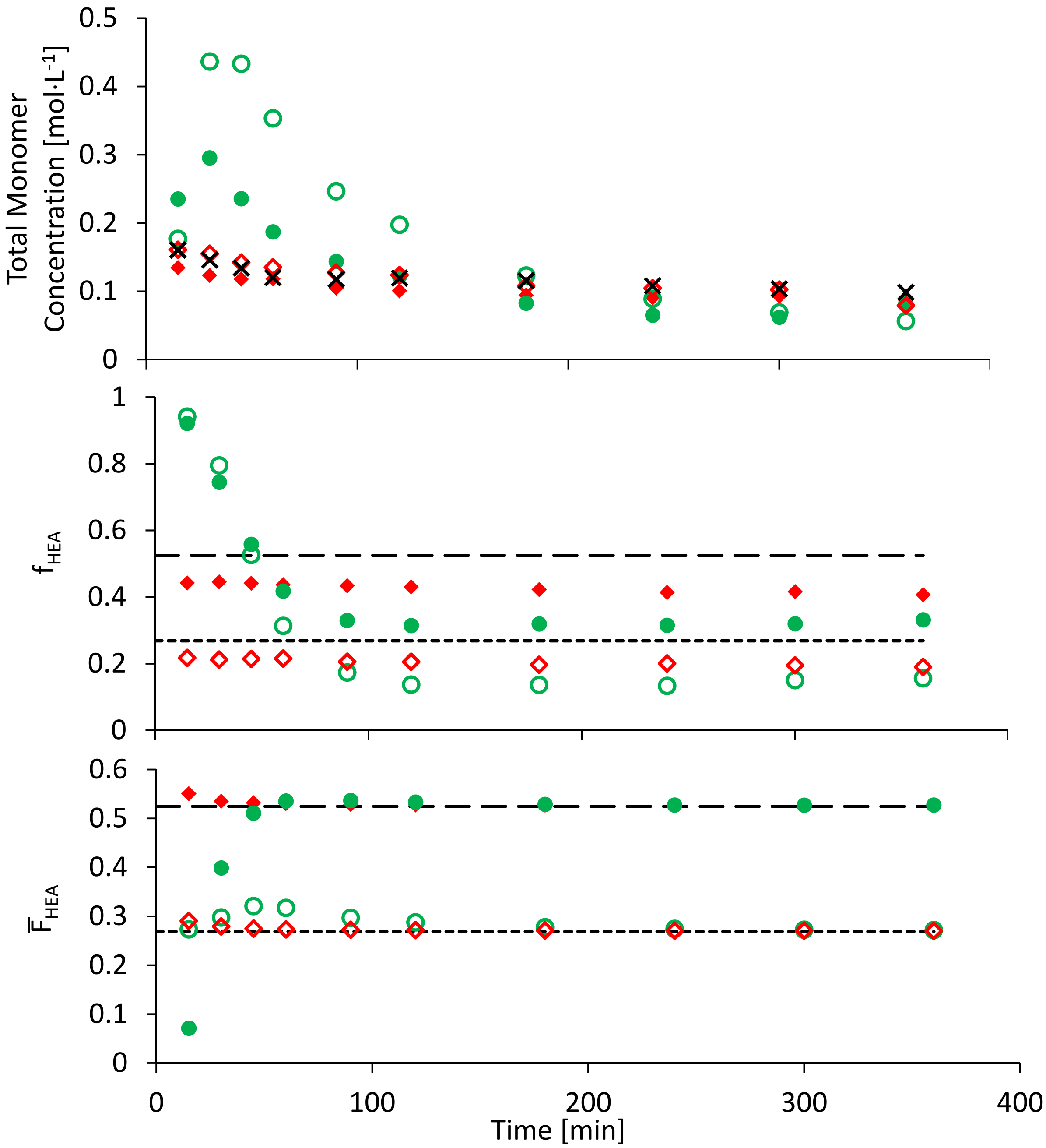

Figure S3: Total free monomer concentration (top), HEA molar fraction in the comonomer (f_{HEA}) (middle) and copolymer (F_{HEA}) (bottom) for BA/HEA semibatch copolymerizations at 138 °C in PeOH (▌) and DMF (▌) with 25 wt% (○, ♢) and 50 wt% (●, ♦) HEA in the comonomer feed. Monomer concentration profile also shown for BA homopolymerization in PeOH (x).

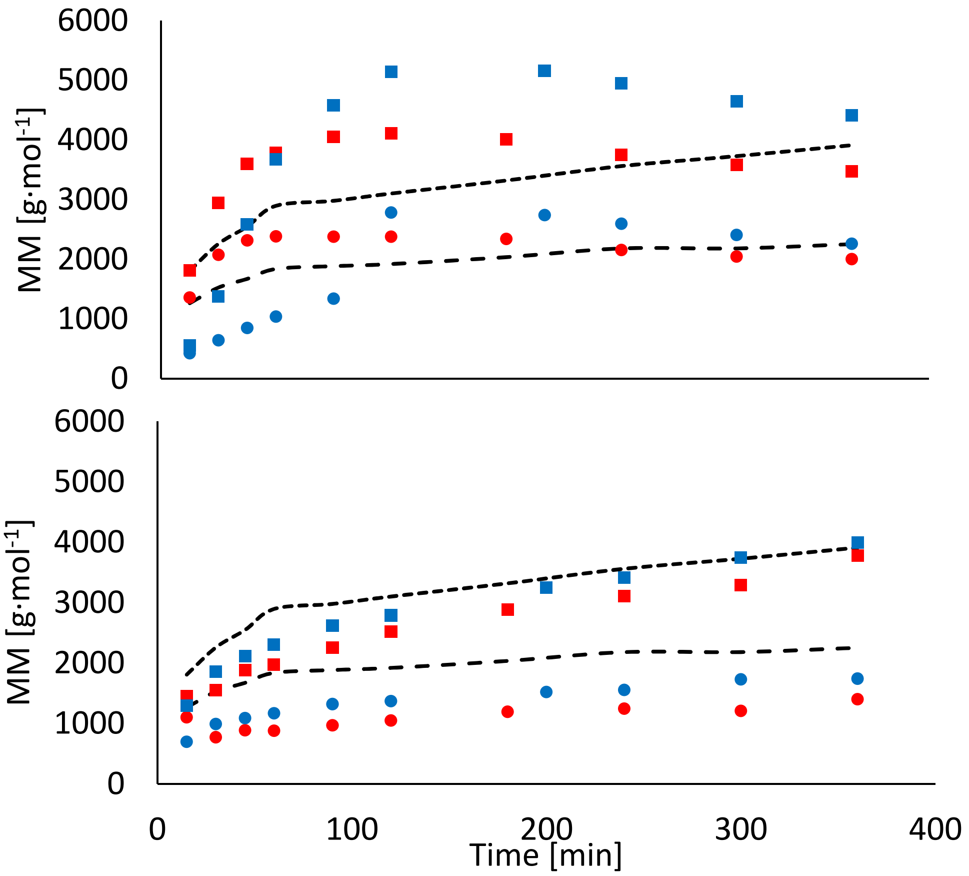

Figure S4: M_n (●) and M_w (■) values determined for BA/HEA copolymers with 25 wt% ( )and 50 wt% ( ) HEA synthesized in PeOH (top) and DMF (bottom) under semi-batch conditions at 138 °C. Lines indicate results for BA homopolymerization in PeOH (M_n = ···, M_w = ---).

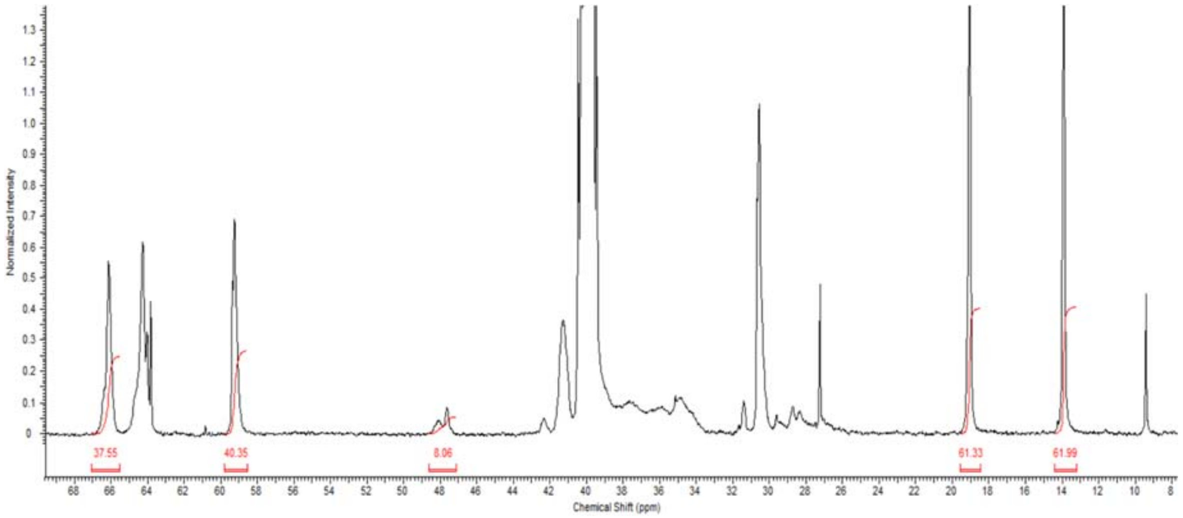

Figure S5: Quantitative ^{13}C-NMR spectrum for BA/HEA copolymer prepared via semi-batch reaction in BPi at 138 °C, with the quaternary carbon peak observable at 48 ppm.

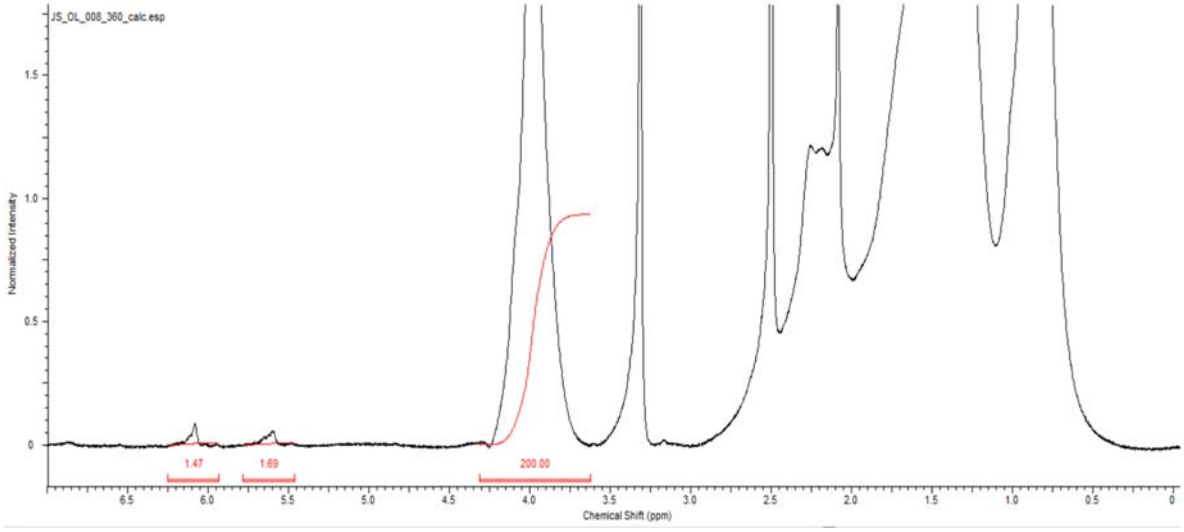

Figure S6: ^{1}H-NMR spectrum for BA homopolymer prepared via semi-batch reaction in BPi at 138 °C, with residual macromonomer signals at 5.6 and 6.1 ppm.
